# Bilingual Mandarin-English preschoolers’ spoken narrative skills and contributing factors: A remote online story-retell study

**DOI:** 10.3389/fpsyg.2022.797602

**Published:** 2022-10-14

**Authors:** Jingdan Yang, Jae-Hyun Kim, Outi Tuomainen, Nan Xu Rattanasone

**Affiliations:** ^1^Department of Linguistics, University of Potsdam, Potsdam, Germany; ^2^Faculty of Arts, University of Groningen, Groningen, Netherlands; ^3^Philosophical Faculty, University of Eastern Finland, Joensuu, Finland; ^4^Macquarie University Centre for Language Sciences, Multilingualism Research Centre, Department of Linguistics, Macquarie University, Sydney, NSW, Australia

**Keywords:** narrative skills, Mandarin-English bilinguals, preschoolers, macrostructure, microstructure

## Abstract

This study examined the spoken narrative skills of a group of bilingual Mandarin–English speaking 3–6-year-olds (*N* = 25) in Australia, using a remote online story-retell task. Bilingual preschoolers are an understudied population, especially those who are speaking typologically distinct languages such as Mandarin and English which have fewer structural overlaps compared to language pairs that are typologically closer, reducing cross-linguistic positive transfer. We examined these preschoolers’ spoken narrative skills as measured by macrostructures (the global organization of a story) and microstructures (linguistic structures, e.g., total number of utterances, nouns, verbs, phrases, and modifiers) across and within each language, and how various factors such as age and language experiences contribute to individual variability. The results indicate that our bilingual preschoolers acquired spoken narrative skills similarly across their two languages, i.e., showing similar patterns of productivity for macrostructure and microstructure elements in both of their two languages. While chronological age was positively correlated with macrostructures in both languages (showing developmental effects), there were no significant correlations between measures of language experiences and the measures of spoken narrative skills (no effects for language input/output). The findings suggest that although these preschoolers acquire two typologically diverse languages in different learning environments, Mandarin at home with highly educated parents, and English at preschool, they displayed similar levels of oral narrative skills as far as these macro−/micro-structure measures are concerned. This study provides further evidence for the feasibility of remote online assessment of preschoolers’ narrative skills.

## Introduction

Children’s early narrative abilities are important for their later literacy skills and play an important role in predicting their general academic performance as well as social and communicative success (Westerveld and Gillon, 2010; Gardner-Neblett and Iruka, 2015; Glisson, 2017; Pinto et al., 2017). Across different languages and cultures, narrative tasks are used as an ecologically valid way of collecting spoken language samples as they provide rich information about children’s language abilities in a naturalistic setting (Botting, 2002; Boerma et al., 2016). For bilingual children, there is a paucity of evidence on the spoken narrative abilities especially for those speaking two typologically distinct languages, such as Mandarin and English. In the United States, Chinese languages (including Mandarin) are spoken by around 2.9 million people at home and are the most frequently spoken home languages other than English and Spanish (United States Census Bureau, 2021). In Canada, Mandarin is one of the most spoken home languages other than English and French (Statistics Canada, 2017). Similarly, in Australia, Mandarin is the most spoken home language other than English (Australian Bureau of Statistics, 2017). Despite the large number of bilingual Mandarin-English communities, little is known about the spoken narrative skills of these bilingual children in each of their two languages. This is especially the case for emerging bilingual preschoolers learning a home language (Mandarin) and a community language (English).

Recently, the COVID-19 pandemic has added to the challenges of testing young children and highlighted the need to move traditional face-to-face testing methods to remote online testing. There is emerging evidence to suggest that remote online testing of child language can be feasible, reliable, and valid (e.g., Sutherland et al., 2017; Manning et al., 2020; Sheng et al., 2021). In this study, the story retell task is used to assess bilingual preschoolers’ spoken narrative skills in each of their two languages to address two aims: First, to add to our understanding on the spoken narrative skills of preschoolers learning two typologically distinct languages (Mandarin vs. English) and, second, to report on factors that predict bilingual preschoolers’ performance on a remote online spoken narrative task.

to document the spoken narrative competence of a group of bilingual Mandarin-English preschoolers, to enrich our knowledge base on bilingual narrative competence in preschoolers learning two typologically diverse languages.

### Spoken narrative skills

Spoken narrative skills, defined as the telling or retelling of a sequence of causally related events, requires the narrator to include detailed information about not only the setting, character, and theme of a story, but also the characters’ actions, emotions, and motivations (Westby, 1991; Glisson, 2017). Spoken narrative skills are evaluated on levels of macrostructure and microstructure. Macrostructure refers to the global organization of a story, consisting of a “setting” plus one or more “episodes.” The “setting” introduces the main character(s) and describes the context of the story (e.g., where the story takes place); and an “episode” includes an initiating event, the character’s goal and attempt in response to the initiating event, and its consequences (Stein and Glenn, 1975; Gillam et al., 2016). Therefore, macrostructure requires adequate higher-level cognitive organization and abilities to conceptualize and plan sequences of events, as well as making inferences about the characters’ motivations to convey a thematically coherent story (Bohnacker, 2016; Rezzonico et al., 2016).

Microstructure, on the other hand, relates to linguistic properties of the narrative in the target language (Stein, 1988; Squires et al., 2014; Bohnacker, 2016; Gillam et al., 2016). The evaluation of microstructure includes not only fine-grained linguistic structures used to construct a coherent narrative discourse, such as specific lexical and grammatical elements (Justice et al., 2010), but also more general measures about the overall spoken language productivity and syntactic complexity in the narrative genre (Westerveld and Gillon, 2010), e.g., total number of utterances, number of words, mean length of utterance (MLU), etc. Microstructures can therefore potentially provide a more detailed profile of a child’s spoken language skills including their strengths and weaknesses in various spoken language domains of morphology, syntax, and semantics (Westerveld and Gillon, 2010).

One of the commonly used methods of eliciting spoken narratives from young children is through a story-retell task. Children are asked to first listen to a story and then reproduce the story, sometimes using visual support (Sheng et al., 2019). With the support of having listened to a prior story script, it is considered less demanding than other narrative tasks, such as story generation, in which children have to construct stories on their own. Therefore, the story-retell task is particularly appropriate for eliciting spoken narratives from younger preschool-aged children and bilinguals (Merritt and Liles, 1989; Westerveld and Gillon, 2010). Over and above the lower task demands, story-retell allows for experimental control over linguistic aspects such as length and complexity in the model story (Pearson, 2002).

### Research on bilingual preschoolers

In terms of macrostructure, it has been suggested that its organization may be universal or invariant across languages (e.g., Berman and Slobin, 1994; Verhoeven and Strömqvist, 2001). Many studies report no differences in macrostructure measures between the two languages of bilingual children (Pearson, 2002; Squires et al., 2014; Bohmacker, 2016; Gagarina et al., 2016; Kunnari et al., 2016; Bonifacci et al., 2018; Méndez et al., 2018), especially for older school-aged children (Pesco and Bird, 2016). More recently, however, Hao et al. (2019) found that Mandarin-English bilingual preschool to school-aged children in the US performed better on “setting” in English than in Mandarin. This could be due to English being the majority/community language leading to better performance compared to the home language (Pesco and Bird, 2016). However, the differences in scores for “setting” were small, suggesting that the macrostructure performance was, in general, still largely similar between the two languages (Hao et al., 2019). It is also unclear whether better English performance would be associated with age as school-aged children receive more formal education, including narrative skills, in English compared to preschoolers.

Microstructure, on the other hand, is more susceptible to variation across bilingual children’s two languages (Pearson, 2002; Uccelli and Páez, 2007; Squires et al., 2014; Hipfner-Boucher et al., 2015; Boerma et al., 2016). This is not surprising given microstructures likely reflect differences in linguistic structures across languages. For example, Spanish-English-speaking 4-6-year-olds showed a strong association among microstructure elements within the same language, but more variation across languages, suggesting that these children are acquiring linguistic structures independently across the two languages (Méndez et al., 2018). On the other hand, in Hao et al. (2019) sample, while microstructure domains of “nominal” and “phrase” showed no significant differences between Mandarin and English, both “modifier” and “verb” were significantly better in English than in Mandarin. The pattern of performance on the various domains also differed within each language. For Mandarin, children were most productive in the “verb” and “nominal” domains, followed by “phrase” and “modifier,” while in English, children produced more “verbs” than the other three domains. In general, these children demonstrated better narrative performance in English than Mandarin, but the differences were larger in *microstructure* than in *macrostructure*, further suggesting that macrostructure is less variable across languages than microstructure (Hao et al., 2019).

### Language experience and bilingual narrative skills

One of the most important sources of influence on language acquisition, apart from general development, is language experience. Earlier age of acquisition and longer use typically lead to better language outcomes (Birdsong, 2009; Bosch et al., 2019; though see Xu Rattanasone et al. (2016) for different length of acquisition effects due to language typology in preschoolers). Bilingual children’s language experience can also vary in terms of amount of language input and use, with both having effects on language development and spoken narrative skills (Hammer et al., 2012; Marchman et al., 2020). Govindarajan and Paradis (2019) found in school-aged children that length of English exposure in school predicted better English narrative skills, but amount of English input (from non-native speakers) and use at home did not predict macrostructure or microstructure abilities in English. Similarly, Hao et al. (2019) found that neither English input or output (production) correlated with performance on English macrostructure or microstructure narrative skills. Given the large age range in their study (4 to 9 years), and the lack of research on bilingual preschoolers’ narrative skills, it is unclear whether these findings would specifically apply to preschoolers. Unlike school-aged children, preschoolers who have not yet received formal education in English (including explicit instructions on narrative skills), are cognitively and linguistically less developed, and therefore their narrative skills might be more influenced by different levels of language input.

### The current study

Although Mandarin is one of the most common home languages around the world, only a few studies have examined the narratives skills of Mandarin-English bilingual children. While Hao et al. (2019) study provided a first important glimpse into the spoken narrative skills of these bilingual children, their study included a sample of children with a wide range of ages from preschool to primary school with varying lengths of exposure to English. This raises questions about the narrative skills of younger preschoolers. Such knowledge will provide us with more insights into early bilingual narrative development and could help inform educators on the language skills of typically developing bilingual preschoolers and their readiness for school.

This study examined a sample of bilingual 3–6-year-olds speaking Mandarin as their home language and learning English as the community language at childcare/preschool. In Australia, children of this age range typically attend a government subsidized private childcare (3–4-year-olds) or a fully funded government preschool (4–5-year-olds) or a kindergarten (5–6-year-olds) with English as the language of instruction. Their narrative skills were examined using a remote online story re-telling task in each of their two languages along with a weekly diary detailing daily Mandarin, English, and Mixed language input, and output for every awake hour. The entire study was conducted through remote online delivery. The following three research questions were addressed.

#### Research question 1

Whether there is a difference between bilingual preschoolers’ macrostructure scores across their Mandarin and English; and whether there are any differences across macrostructure elements within each language. We predict a positive correlation between Mandarin and English, and no significant differences in overall performance on specific macrostructures across languages, but performance levels on macrostructure elements within languages might vary (Hao et al., 2019).

#### Research question 2

What are bilingual preschoolers’ spoken narratives skills in terms of microstructures across their two languages? Is there a difference between bilingual preschoolers’ microstructure domain scores in Mandarin and English? Within each language, are there any differences in the production of individual microstructure domains? We predict that there could be no correlations cross-language in microstructure domains (unlike macrostructure; Hao et al., 2019). For Mandarin, performance should be best in the “verb” and “nominal” domains, followed by “phrase” and “modifier,” and for English, performance should be best on “verbs” compared to the other three domains (Hao et al., 2019).

#### Research question 3

Are there any associations between preschoolers’ narrative performance (macro/microstructural) and various contributing factors, such as chronological age, length of language exposure, and language input and output? We predict that chronological age would correlate positively with macrostructure (general developmental effect) and length of language exposure would correlate positively with microstructure (linguistic experience effect; Hao et al., 2019). We further predict that language input and output would correlate with performance in both languages (preschoolers are not yet receiving systematic schooling on narrative skills in English, unlike the school-aged children in the Hao et al., 2019).

## Materials and methods

### Participants

A total of 25 Mandarin-English (ME) bilingual preschoolers participated in this study but 5 were excluded for not switching languages, i.e., produced both stories only in English (*N* = 1) or only in Mandarin (*N* = 2), or could not finish either story (*N* = 2). The final sample consisted of 20 children (15 girls and 5 boys) aged between 3;10 (year; month) and 6;4 [mean age = 4;11, Standard Deviation (SD) = 8.7 months]. The primary carers of these children were in general well educated with two having received vocational training, eight completed an undergraduate degree and 10 postgraduate degrees. Of these primary carers 14 have received their highest level of training in Australia using English and 6 in China using English (2) or Mandarin (4). All participants resided in Sydney, Australia at the time of testing. Ethical approval for remote online testing was only allowed at the time and obtained from Macquarie University (approval number: 52021662724256).

Each child’s primary caregiver was asked to complete a demographic information and language history questionnaire (see Appendix A). All children were raised in Mandarin-speaking households with native Mandarin-speaking parents (only one parent grew up speaking Cantonese and English). All parents were born in China (mainland or Hong Kong). The children’s average age of acquisition (AoA) for English was 22 months (SD = 8.5 months; range: 11–41 months). All children were exposed to English through childcare before the age 36 months, except for one child at 41 months. The average length of English exposure was 37 months (SD = 12.7 months, range: 20–62 months). No language disorder or hearing impairment was reported.

### Materials

Each child completed two story-retell tasks, one in English and one in Mandarin. Two different sets of wordless story pictures designed to elicit age-appropriate languages abilities in each language were used to avoid practice effects. The English picture story was *Ana gets lost* (Westerveld and Gillon, 2010), for children 4;0 to 7;6. The Mandarin story was taken from the “学龄前儿童语言能力测试 [Language Proficiency Test for Preschool Children]” [天津师范大学语言研究所 (Tianjin Normal University), 2016], for children 3;0 to 7;0. The stories were pre-recorded by a female native Australian English speaker and a native Mandarin speaker.

The primary caregiver of each child also provided a 7-day diary of hourly activities children were engaged in, interlocutors, if any, and language(s) heard (input) and produced (output) by the child. The diary data was later coded into total hours of hearing and speaking Mandarin, English, or both languages (mixed). The percentages of input and output of each language were then calculated by dividing the total number of hours hearing or speaking that language with the total number of awake hours.

The diary data is summarized in Table 1. Since the percentages of input to and output from children in each language were similar within languages, a single measure for each language was derived by averaging across input and output for that language. This new measure was used as a general indicator of language experience for each language. A paired *t*-test conducted between the average percentages of English and Mandarin language experience score found that children had significantly more experience in English (Mean = 47%) than in Mandarin (Mean = 29%). The mixed language experience data (both input and output) was not included as we were only interested in pure English and Mandarin language experiences (mixed language would include both languages therefore masking the independent effect of each language).

**Table 1:** Means, standard deviations (SD) and range for percent of input and output in each language.

Measure	English			Mandarin			Mixed		
	Mean	SD	Range	Mean	SD	Range	Mean	SD	Range
Input	47%	13%	27–70%	30%	19%	2–58%	23%	19%	0–62%
Output	48%	14%	20–76%	27%	20%	1–58%	25%	20%	0–61%
Average	47%	13%	24–73%	29%	19%	2–58%	24%	19%	0–59%
t-test	*t* = 3.11, *p* < 0.01, Cohen’s d = 0.70								

### Procedures

#### Elicitation

The data collection was done during the COVID19 pandemic, as a result, the testing took place *via* remote online delivery using Zoom. Children were invited to attend Zoom meetings with camera on and in the company of their parent(s). Bilingual Mandarin-English-speaking research assistants were trained to administer the tests *via* Zoom. Instructions were given to parents that they need to allow their child to complete the tests independently without any help, but they could encourage their child to pay attention to the task. It was also explained to each child and their parent that they were not expected to remember every detail of the story.

The tasks were administered first in Mandarin and then in English. The order of presentation was not counterbalanced as the study was conducted remotely online, in the children’s homes, with help from their Mandarin-speaking parents and so all sessions began in Mandarin. Also, having their parents encourage them to participate in the home language helped ensure better engagement from the child participants. In the beginning of the story-retell task, children were instructed to carefully listen to a story. A set of wordless pictures appeared on screen one by one with the audio-recording of the story. After the story had finished, the same pictures were presented to the children again and they were asked to retell the story in their own words. If children did not start retelling the story spontaneously, prompts (e.g., “What happened in the beginning?”) were used to help elicit responses. Parents were asked not to provide answers or repeat the answers. During each session, the instructions were given to children only in the target language. For children who could not complete the story-retell in one session, another Zoom session was arranged (*N* = 5).

#### Transcription

The recordings of children’s story-retell were transcribed by a ME bilingual speaker (the first author) in ELAN (Max Planck Institute for Psycholinguistics, 2021) according to the CHAT transcription format (MacWhinney, 2019). Following the previous convention used in Hao et al. (2019), all task-related speech produced by the child (in forms of sentences, clauses, phrases, or single words) were segmented into communication units (C-unit), which is a main clause with its modifiers (Loban, 1976). Within each C-unit, transcription was done at the word-level for both Mandarin and English: Mandarin narratives were transcribed into written Chinese characters and English narratives into written English words. Verbal instructions from the experimenter, interventions from parents/caregiver, or task-unrelated speech or non-speech sounds (sighs, sneezes, coughs, crying, laughing, etc.) produced by the child were excluded from transcriptions. Inter-rater reliability was conducted between the first author and the last author (also a ME bilingual speaker) on 10% of the recordings in both Mandarin and English. Inter-rater agreement was 73.3% for C-units coded across the two raters (kappa = −0.134, *z* = 0.974, *p* = 0.330) suggesting substantial to high agreement (McHugh, 2012). On closer examination, the mean percent of disagreement across all C-units transcribed was 7.3% between the two raters (i.e., mean number of disagreements/number of agreements + number of disagreements per C-unit).

#### Coding and scoring

The evaluation included macrostructure and microstructure analyses. For macrostructure analysis in English, we chose the Story Quality Rubric (Westerveld and Gillon, 2010) as it was originally designed to analyse the macrostructure for “Ana gets lost.” The decision was also made based on the consideration that our participants were younger than those in Hao et al. (2019) study, and their narrative productions were much simpler. Therefore, using other more complex rubrics such as Monitoring Indicators of Scholarly Language (MISL; Gillam et al., 2016) is likely to lead to overall poor performances (a floor effect). For macrostructure analysis in Mandarin, the rubric was adapted from the English version. Both macrostructure rubrics contained eight elements: *Introduction, Theme, Main Character, Supporting Character(s), Conflict, Coherence, Resolution and Conclusion.* Based on different levels of completion, each child was awarded different points for each element: 5 points if the child showed proficient ability in supplying the required details, 3 points if the child showed emerging ability in providing some details, and 1 point if the child provided minimal or no information. The scores were summed up to yield a total macrostructure score for each language. The possible minimum score was 8 and the maximum score 40. Details about the macrostructure scoring criteria for English and Mandarin can be found in Appendix B,C.

For microstructure analysis, we analysed the language samples for both general and fine-grained microstructures. Four general microstructure measures were evaluated to provide information about children’s general narrative skills: total number of words (TNW), number of different words (NDW), total number of C-units (TNC), and MLU in words (MLUw). Data were extracted in CLAN (MacWhinney, 2000) with the “*freq*” and “*mlu -t%mor*” commands.

For the measures of fine-grained microstructures, we modified the Narrative Assessment Protocol (NAP) by Justice et al. (2010). Four domains of language (*phrase structure*, *modifier*, *nominal*, and *verb*) were evaluated, and each contained four to six elements. The English microstructure rubric contained 10 elements in four domains: *Phrase* (passive structure/locative phrase/temporal phrase); *Modifier* (adjective, adverb, negation); *Nominal* (personal pronoun), and *Verb* (copula/irregular past tense/regular past tense). The rubric used for Mandarin microstructure analysis was an analogous rubric to the English version. Items that do not have analogous structures in Mandarin (e.g., English verb inflections) were excluded; unique features of Mandarin grammar (e.g., “ba” structure) were added. The final Mandarin rubric contained the same number of elements under four domains: *Phrase* (“ba” structure, locative phrase, temporal phrase); *Modifier* (adjective, adverb, classifier); *Nominal* (personal pronoun) and *Verb* (progressive aspect marker, perfective aspect marker, resultative aspect marker). However, unlike Hao et al. (2019), we did not include the Mandarin passive, “bei” structure, in our rubric. The Mandarin story we used, developed specifically for Mandarin speaking preschoolers, did not have passive structures. Indeed, Yip and Matthews (2007) showed that Cantonese-English simultaneous preschoolers (acquiring both languages from birth) did not produce ‘bei’ or passives in general with high frequency (Cantonese is a closely related Sinitic language to Mandarin). See Appendix D,E and for the English and Mandarin detailed microstructure rubrics.

Following Hao et al. (2019), for each microstructure element, we used both the 0–3 scale frequency score as in the NAP (Justice et al., 2010) and the raw frequency score. For 0–3 frequency score, each element was given a maximum score of three (even if the occurrence was more than three). The raw frequency considers all occurrences of an element and reflects children’s productivity on that element. For example, if an element occurred 4 times, it was scored 3 for the 0–3 frequency score and 4 for the raw score. Consistent with NAP, for *Modifiers*, *Nominals*, and *Verbs*, only unique usages (types) were counted; but unique usage was not required for scoring *Phrasal* elements (tokens). Since there was only one unique personal pronoun in the Mandarin story, and the production of classifiers was limited, we scored tokens instead of types for these two elements. Only accurate productions of microstructure elements were counted.

#### Statistical analysis

To answer the first and the second research questions about differences in bilingual ME children’s macro/microstructures between Mandarin and English, and among individual macrostructure elements/microstructure domains within each language, Linear mixed effects models were fitted using the lme4 package (Bates et al., 2015) in R (R Core Team, 2021). Following Hao et al. (2019), language experience was included in the models as a covariate. Mandarin language experience was subtracted from English, generating a difference score for each participant, which was then entered into the models as a covariate for both macrostructure and microstructure analyses (mean difference score = 19%; SD = 27%; range: −32 – 64%).

Regarding the fixed effect(s), for the *macrostructure* analysis, only “language” (Mandarin vs. English) was included in the model, however for the *microstructure* analysis, both “language” and “domain” (*phrase structure*, *modifier*, *nominal*, & *verb*) were included in the model. Varying intercepts were fitted for participants as random effects. The same linear mixed effects model was successively fitted for every macrostructure element/microstructure domain. For within language comparisons, pair-wise *t*-tests with Bonferroni adjustments (Benjamini and Hochberg, 1995) were conducted between macrostructure elements/microstructure domains of each language.

The third research question was whether there were associations between children’s narrative performance (macro/microstructural) and various contributing factors. Considering the relatively small sample size, the non-parametric Spearman correlation test was conducted on narrative performance scores (macrostructure English/macrostructure Mandarin/microstructure English/microstructure Mandarin, abbreviated as mac_eng / mac_man / mic_eng / mic_man respectively), chronological age (Age), age of acquisition for English (AoA), length of English exposure (E_length), language experiences (average of input and output) in English/Mandarin condition (E_Exp / M_Exp), and parent’s bilingual dominance scores (Bilingdom; derived from the language history questionnaire). The Benjamini–Hochberg procedure (Benjamini and Hochberg, 1995) was conducted to avoid false discovery from multiple tests.

## Results

### Macrostructure

First, regarding cross-language comparisons, macrostructure total scores did not differ significantly, suggesting that overall performance across the two languages did not differ (Table 2). In terms of individual macrostructure elements, participants differed only on “Main character” and “Supporting character” across the two languages, with better performances in Mandarin than in English.

**Table 2:** Parameter estimates for between language (Mandarin vs. English) comparisons on macrostructure.

Measure	English	Mandarin	*F*	*p*
	Mean (SD)	Mean (SD)		
Introduction	2.60 (1.67)	2.40 (1.60)	0.192	0.666
Theme	3.40 (1.90)	4.20 (1.01)	2.823	0.101
Main character	3.30 (1.63)	4.40 (1.31)	**6.066**	0.024^*^
Supporting character(s)	3.10 (1.52)	4.20 (1.20)	**6.449**	0.015^*^
Conflict	2.30 (1.49)	2.80 (1.82)	1.508	0.235
Coherence	2.50 (1.70)	2.40 (1.47)	0.045	0.834
Resolution	3.70 (1.49)	4.10 (1.02)	1.000	0.330
Conclusion	3.50 (1.28)	3.60 (0.94)	0.137	0.716
Total	24.40 (8.63)	28.10 (5.03)	4.135	0.056

Significant results are in bold. ^*^p < 0.05.

In terms of within-language comparisons, in both English and Mandarin, children scored higher on “Theme,” “Main character,” “Supporting character,” “Resolution” and “Conclusion” than on “Introduction,” “Conflict” and “Coherence” (see Appendix F for pairwise *t*-tests), again showing similar patterns of performance across the two languages.

### Microstructure

#### General microstructures

The general microstructure scores are presented in Table 3. The properties of the model stories are shown below under the “Model story” column. A “proportion” column indicates proportion of model-like structures produced by the children in relation to the target story. The Mandarin story was relatively simpler than the English story with fewer total C-units, total words, and different words, whereas the MLU in words (MLUw) was similar between the two languages. The descriptive data suggest that, as compared to the target story heard by the children, our participants produced relatively shorter narratives, with smaller numbers of TNC, TNW and NDW. They did however produce more model-like structures on each measure compared to the target story they heard for Mandarin than English.

**Table 3:** Means, standard deviations (SD), and ranges on general microstructure measures of child productions compared to model stories in English and Mandarin.

Measure	English					Mandarin				
	Mean	SD	Range	Model Story	Proportion%	Mean	SD	Range	Model Story	Proportion%
TNC	11.57	5.06	3–20	**24**	48.75	8.19	3.08	4–14	**12**	68.25
TNW	82.76	44.87	13–175	**193**	42.88	54.90	21.71	26–105	**118**	46.53
NDW	43.05	20.50	10–87	**115**	37.44	32.52	9.53	20–55	**63**	51.62
MLU-w	5.93	1.92	2.00–8.89	**8.04**		6.49	1.32	4.50–9.17	**9.83**	

The scores presented were averages of the two stories. TNC refers to total number of C-units; TNW refers to total number of words; NDW refers to number of different words; and MLUw refers to mean length of utterances in words. Significant results are in bold.

#### Fine-grained microstructures

The 0–3 frequency scores and raw frequency scores of each microstructure element in two languages are summarized below in Table 4 (see Appendix G for proportion of model-like structures produced). The domain scores were derived by averaging across all elements within each domain, as shown in Table 4 (i.e., the mean and standard deviation of English Modifier elements across all participants were 1.75 and 1.12, respectively).

**Table 4:** Within language analysis of microstructure across domains for English and Mandarin in frequencies (0–3 and raw frequency).

Domain	English			Mandarin		
	Elements	Mean (SD)		Elements	Mean (SD)	
		0–3 frequency	Raw frequency		0–3 frequency	Raw frequency
Modifier	Adjective	2.05 (1.23)	2.45 (1.82)	Adjective	1.95 (1.00)	2.05 (1.19)
	Adverb	1.85 (0.99)	2.25 (1.62)	Adverb	2.10 (1.12)	2.45 (1.54)
	Negation	0.55 (0.60)	0.55 (0.60)	Classifier	1.90 (0.97)	1.90 (0.97)
	**Average**	**1.48 (0.74)**	**1.75 (1.12)**	**Average**	**1.98 (0.69)**	**2.13 (0.85)**
Nominal	Pronoun	2.15 (1.04)	2.80 (1.82)	Pronoun	2.30 (1.03)	4.00 (2.96)
	**Average**	**2.15 (1.04)**	**2.80 (1.82)**	**Average**	**2.30 (1.03)**	**4.00 (2.96)**
Phrase	Locative phrase	1.10 (1.21)	1.40 (1.82)	Locative phrase	1.45 (1.10)	1.45 (1.10)
	Passive phrase	0.35 (0.49)	0.35 (0.49)	Ba structure	0.60 (0.60)	0.60 (0.60)
	Temporal phrase	1.00 (1.08)	1.10 (1.29)	Temporal phrase	0.35 (0.49)	0.35 (0.49)
	**Average**	**0.82 (0.69)**	**0.95 (0.97)**	**Average**	**0.80 (0.45)**	**0.80 (0.45)**
Verb	Copula & Auxiliary	1.60 (1.19)	1.7 (1.38)	Perfective aspect marker	2.35 (0.81)	3.55 (2.16)
	Irregular past tense	2.15 (1.14)	2.80 (1.96)	Progressive aspect marker	0.55 (0.60)	0.55 (0.60)
	Regular past tense	1.15 (1.27)	1.60 (2.16)	Resultative aspect marker	2.15 (0.99)	2.55 (1.50)
	**Average**	**1.63 (1.05)**	**2.03 (1.68)**	**Average**	**1.68 (0.59)**	**2.22 (1.09)**
Total			**17.00(0.87)**			**19.45(1.25)**

Significant results are in bold.

For mean 0–3 frequency scores in each domain, the results showed a significant main effect of domain [*F* (3, 57) = 32.48, *p* < 0.001], but they did not show a significant main effect of language [*F* (1, 133) = 2.74, *p* = 0.100] or interaction between language and domain [*F* (3, 133) = 1.24, *p* = 0.298]. Pairwise comparisons showed that all domains in English differed significantly with each other except between “Modifier” and “Verb” (Nominal > Modifier = Verb > Phrase), and all domains in Mandarin differed significantly with each other except between “Modifier” and “Nominal” domains (Nominal = Modifier > Verb > Phrase). See Appendix H for the pairwise comparisons.

### Factors influencing narrative development

As shown in Table 5, no significant correlations were found between English and Mandarin for macrostructures or microstructures. Within each language, while there was a strong positive correlation between macrostructure and microstructure in English (r_s_ = 0.799, *p* = 0.002), macrostructure and microstructure did not reach significance for Mandarin.

**Table 5:** Correlation matrix of narrative skills with contributing factors.

	mac_eng	mic_eng	mac_man	mic_man	Age	AoA	E_length	Bilingdom	E_exp	M_exp
mac_eng		0.799^**^	0.469	0.146	0.692^*^	0.049	0.50000	0.471	−0.084	−0.399
mic_eng			0.416	0.379	0.5790	0.342	0.25000	0.302	0.105	−0.558
mac_man				0.518	0.711^*^	0.073	0.40900	0.311	−0.264	−0.228
mic_man					0.2170	0.477	−0.14100	0.414	−0.346	0.079
Age						−0.078	0.734^**^	0.516	−0.075	−0.265
AoA							−0.690^*^0	−0.142	0.034	0.085
E_length								0.473	−0.145	−0.212
Bilingdom									−0.404	0.040
E_exper										−0.237
M_exper										

E: English; M: Mandarin; macro: macrostructure total score; micro: microstructure 0–3 frequency total score; Age: chronological age; AoA: English age of acquisition; E_length: length of English exposure; Bilingdom: parent’s bilingual dominance score; E_Exp/M_exp: average percent of language input and output in English/Mandarin. ^*^p < 0.05, ^**^p < 0.001, indicates a significant correlation after the Benjamini–Hochberg procedure.

Regarding potential contributing factors, chronological Age was significantly and positively correlated with macrostructures in both English (r_s_ = 0.692, *p* = 0.012) and Mandarin (r_s_ = 0.711, *p* = 0.012), but not with microstructures. English Age of Acquisition had no significant correlations with any narrative measure in either language. Length of English exposure, although showing a significant positive correlation with Age and negative correlation with Age of Acquisition, showed no significant correlation with either of the narrative measurements. Language experience in general did not significantly correlate with any narrative measures.

## Discussion

Using remote online assessment, this study investigated Mandarin and English (ME) learning bilingual preschoolers’ spoken narrative skills in terms of macrostructure and microstructure within and across languages. Potential factors influencing narrative development were also explored. ME bilingual preschoolers demonstrated similar narrative skills in their two languages, in both our measures of macrostructure and microstructure. More cross-linguistic differences were found in microstructure than macrostructure. Age was significantly positively correlated with macrostructures in both languages. Language experience, however, had no significant correlations with any aspects of children’s narrative skills. These results were generally consistent with the findings from previous face-to-face studies (as outlined below), suggesting that it is feasible to use remote online spoken narrative tasks to measure bilingual preschoolers’ spoken narrative skills.

### Bilingual narrative skills

#### Macrostructure

The first research question investigated whether there was any difference between bilingual ME preschoolers’ macrostructure scores across languages, and whether there were any differences among various macrostructure elements within each language. We found that the overall performance on macrostructure measures did not differ between the two languages. Within both Mandarin and English, the preschoolers scored higher on “Theme,” “Main character,” “Supporting character,” “Resolution,” and “Conclusion” than on “Introduction,” “Conflict,” and “Coherence.” These findings are generally consistent with previous studies (Bohnacker, 2016; Fiestas and Peña, 2004) and provide further support for children exhibiting different levels of ability across the different macrostructure elements at various stages of development.

However, there were differences in “Main character” and “Supporting character” elements between the two languages. For these two elements, children scored higher in Mandarin than in English. This is likely a reflection of the uneven complexity of the two stories used. In the Mandarin story, designed specifically for Mandarin-speaking preschoolers, the main character (the little rabbit) was introduced alone in the first sentence; while in the English story, the main character (Ana) was introduced along with two supporting characters (mom and dad). Regarding the supporting character(s), there was only one in the Mandarin story (the little hedgehog), whereas there were several in the English story (mom, dad, big brother Tom, & the policeman). Therefore, the cross-language differences that we observed in the “Main character” and “Supporting character” are likely due to the differences of the two stories used to elicit narratives. Apart from these two elements, there were no significant cross-language differences on macrostructure, which aligns with previous findings that macrostructure reflects the global story organization and relies more on general cognitive abilities, so it tends to be less variable across languages (e.g., (Bohnacker, 2016; Rezzonico et al., 2016).

#### Microstructure

The second research question investigated any differences in bilingual ME preschoolers’ microstructure scores between their Mandarin and English, and within each language. We predicted that microstructure was less likely to show cross-language similarities than macrostructure. But children might show different levels of ability on different microstructure domains within each language, due to the different linguistic characteristics of Mandarin and English. The results showed there were no significant interactions between language and domain, or on the main effect of language, indicating that our bilingual preschoolers performed similarly on their two languages. Our results differ from the commonly reported findings that microstructure usually differs across languages and is more variable than macrostructure (e.g., Pearson, 2002; Justice et al., 2010; Boerma et al., 2016). Indeed, Hao et al. (2019) showed different patterns for children’s productions in Mandarin and English. However, it is possible that our sample of preschoolers have not yet developed enough linguistic competence or vocabulary in each language to show language-specific effects in spoken narrative skills. The different patterns across these two studies could indicate differences in stages of narrative development, and we had a much tighter sample of preschoolers who were predominantly 4- and 5-year-olds as opposed to the school-aged sample in Hao et al. (2019). The different results between our study and Hao et al. (2019) could also reflect difference in types of bilinguals. Our preschoolers were more akin to simultaneous bilinguals while Hao et al. (2019) sample of children had wider ages and ages of exposure to English, reflective of a mix of both simultaneous and early child L2 learners. Future studies should consider separating simultaneous and sequential bilinguals, as well as preschoolers from school aged children who are likely to be receiving structured reading and writing instructions in English (Whitehurst and Lonigan, 2001; Reese et al., 2010).

While our study did not indicate that our preschoolers were more dominant in English, Hao et al. (2019) study did show that their sample performed better in English than Mandarin, with greater language-specific knowledge in English. According to the authors, the significantly larger proportion of language experience in English (above 60%) than in Mandarin was responsible for driving the different performance across languages. Our study differs from Hao et al. (2019) in several ways. First, our participants were younger than those in Hao et al. (2019) study, who were exposed to English for longer with many receiving structured reading and writing instructions at school. Second, while our sample of preschoolers also had more language experience in English (mean = 47%) than in Mandarin (mean = 29%), the difference was not as large as in Hao et al. (2019) sample, i.e., 66–34% input and 75–25% output. It might be possible that smaller differences between home and community language experiences in our sample were not large enough to drive cross language differences in microstructures. Therefore, both developmental and language experiences might be driving different findings across the two studies which needs to be teased apart in any future research.

#### Factors influencing bilingual narrative development

The last research question relates to the effects of various factors that might contribute to ME preschoolers’ narrative performances. Consistent with Hao et al. (2019), we found no direct relationship between language experiences (input and output) and macrostructure/microstructure in either Mandarin or English. This shows that despite some children receiving more English than Mandarin, this difference did not have any relationship with their spoken narrative skills in either language. The range in difference scores between Mandarin and English in our sample were not restricted in range and should be sufficiently large enough across the sample to show a relationship with spoken narrative skills if one exists: from −32% (more Mandarin) to +64% (more English). However, our sample also reported on average 20% Mixed language input and output (30% for Mandarin only and 50% for English only). Perhaps more needs to be understood about the role of mixed language output to better account for the language experience of these bilingual preschoolers, e.g., by using more intrusive and time intensive data collection methods such as audio and video recordings.

In terms of other factors, as predicted, older children in general showed better macrostructure performances than younger children in both languages; but age did not affect their microstructure performances. This suggests that macrostructure skills probably develop more rapidly during preschool than microstructure and is therefore easily detectable and affected by development. However, different from Hao et al. (2019), both age and length of English exposure failed to show any relationship with spoken narrative performance measures in either language. This might be due to restricted ranges in a sample of predominantly 4- and 5-year-olds.

### Limitations and future directions for remote online testing

One limitation is that the English story was slightly more complex in macrostructure than the Mandarin story. Children in general supplied below 50% of the model story in C-units and word tokens and types for the English story, but between 50 and 60% for the Mandarin story. This might suggest that the English story was too challenging for these bilingual preschoolers. In addition, given that MLUw was similar across the two stories and the patterns of performance were similar across the different microstructure domains and in both languages, the differences between the stories did not affect our purpose of making crosslinguistic comparisons. However, both the English and Mandarin stories lacked enough variety of certain microstructure elements, e.g., nominal items. As a result, only one element, personal pronouns, was included in the Nominal domain, making the overall microstructure evaluation less representative and comprehensive. The Multilingual Assessment Instrument for Narratives (MAIN), now with both Mandarin and English versions available, could be a good alternative all-in-one assessment for ME preschoolers (Gagarina et al., 2019; Luo et al., 2020).

Second, the language experience data collected from caregivers were inconsistent from child to child. Although we provided a template for documenting children’s language activities and asked caregivers to provide as much detail as possible, some failed to conform to these instructions. Future studies should consider whether other methods of collecting real-time parental reports of diaries (e.g., sending text alerts) or samples of audio/video recordings might be more effective ways of collecting language experience data, albeit more intrusive and time intensive.

Third, the sample is relatively homogenous with limited age and ranges of English exposure. While this met the purpose of this study, i.e., examining bilingual preschoolers’ spoken narrative skills, future studies should consider comparing preschoolers with school aged children or using longitudinal designs to capture developmental changes. Related to this, we did not examine the relationship between spoken narrative skills and individual variation on general cognitive abilities or combined vocabulary. This may be of interest for future studies to better understand individual variation (including sex) on macrostructures (global cognitive abilities) and microstructures (linguistic specific abilities) in dual language development. Future studies could also consider comparing similar children with their monolingual counterparts and school aged L2 learners to better understand effects of age of language acquisition (see Meisel, 2018). Such studies could also examine errors children make in each language to better understand the effect of language typology on dual language acquisition. For example, spoken Mandarin does not have gender pronouns (e.g., he/she) nor a case system (e.g., he/him). Mandarin is also an isolating language with no nominal (plurals) and verbal (tense) inflections. However, given that our sample is exposed to English as the community language very early in their development as preschoolers, we might expect this population to acquire structures in English similar to their monolingual peers (Meisel, 2004, 2009, 2018).

Finally, the global COVID19 pandemic has highlighted the need for more remote online research. While this study has provided some confidence in using remote online testing for assessing bilingual preschoolers’ spoken narrative skills, more work is needed to evaluate the reliability and validity of remote online narrative elicitation tasks. One challenge we did experience is with managing parental intrusion, which was also reported in Du et al. (2020). When testing young children *via* remote online assessment, parent support is essential to enable the success of the session, however managing parental intrusion is much more difficult when parents must stay with their children to troubleshoot unexpected technical challenges. Our experience is that, for some parents, despite explicit instructions to encourage rather than provide linguistic support to their children, they continued to provide prompts despite explicit instructions at the beginning of each session and requests throughout the session to only provide encouragement. This would be especially disruptive in clinical settings. Such intrusions are also reported with similar sample of children (Du et al., 2020) and easier to manage in face-to-face testing where parents can wait in the same room but with some distance away from the child to avoid direct interference. Another challenge we experienced is reduced quality of some of the audio recordings due to not having control over the recording environment, e.g., children speaking too softly, poor internet connection, children who are unable to sit still, construction noise, etc. Coding these recorded productions offline can be challenging especially for untrained coders. While word level transcriptions can be made, acoustic analysis of segments could not be reliably conducted. One solution might be to send portable recorders to parents with detailed (written/video) instructions and the use of apps that indicate level of environmental noise (e.g., the ListenApp from Macquarie University).

## Conclusion

Using remote online testing, this study investigated bilingual ME preschoolers’ narrative skills at the levels of macrostructure and microstructure, both cross-linguistically and within-languages. The findings contribute to better understanding of the typical spoken narrative skills of ME preschoolers. It has provided further support that macrostructure develop similarly across the two languages, even during very early bilingual development in preschoolers. The age effect further suggests that cognitive development might be important in driving macrostructure development in narrative skills. The lack of effect of age of exposure to English for microstructures and lack of relationship between cross-linguistic experience on spoken narrative skills for ME speaking preschoolers may be an effect of typological distance between the two languages being acquired, but this needs further investigation. These findings add to our understanding of typical bilingual development and suggest that despite differences between home and community language experiences, it is possible for children’s dual language development in spoken narrative skills to be largely balanced.

## Data availability statement

The raw data supporting the conclusions of this article will be made available upon request.

## Ethics statement

The studies involving human participants were reviewed and approved by Macquarie University Humanities and Social Sciences (HASS) Human Research panel (approval number 52022662736215). Written informed consent to participate in this study was provided by the participants’ legal guardian/next of kin.

## Author contributions

JY wrote this thesis as part of her European Master’s in Clinical Linguistics (EMCL+) and was responsible for transcribing the data, performing data analyses, interpretation of the results and drafting the manuscript. All authors contributed to the article and approved the submitted version.

## Funding

This project received funding from Macquarie University Centre for Language Sciences.

## Conflict of interest

The authors declare that the research was conducted in the absence of any commercial or financial relationships that could be construed as a potential conflict of interest.

## Publisher’s note

All claims expressed in this article are solely those of the authors and do not necessarily represent those of their affiliated organizations, or those of the publisher, the editors and the reviewers. Any product that may be evaluated in this article, or claim that may be made by its manufacturer, is not guaranteed or endorsed by the publisher.
