# Supplementary figures and images for: Bilingual Mandarin-English preschoolers’ spoken narrative skills and contributing factors: A remote online story-retell study
Source: Front Psychol. 2022 Oct 14;13:797602. doi: 10.3389/fpsyg.2022.797602 (PMC9615547; doi:10.3389/fpsyg.2022.797602)

**Appendix A. Language history questionnaire**

**
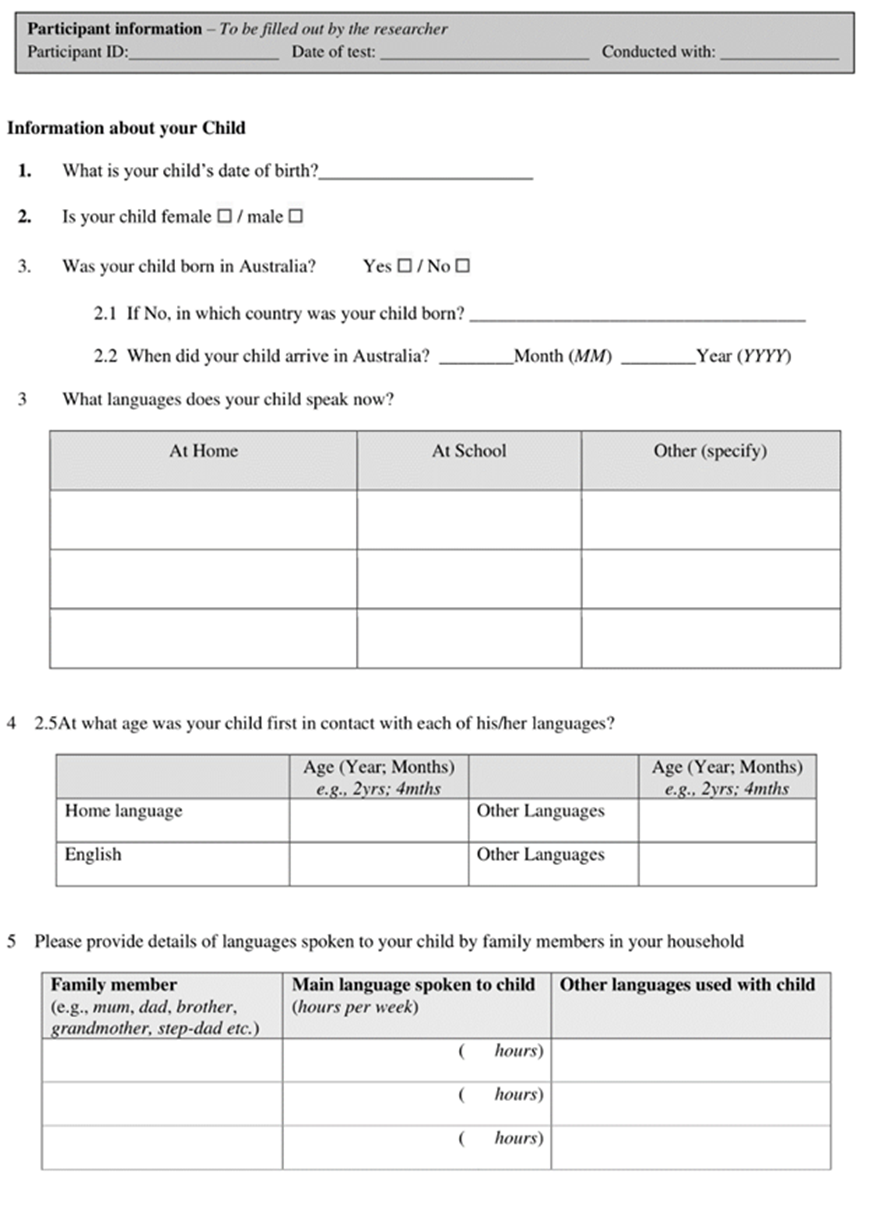
**


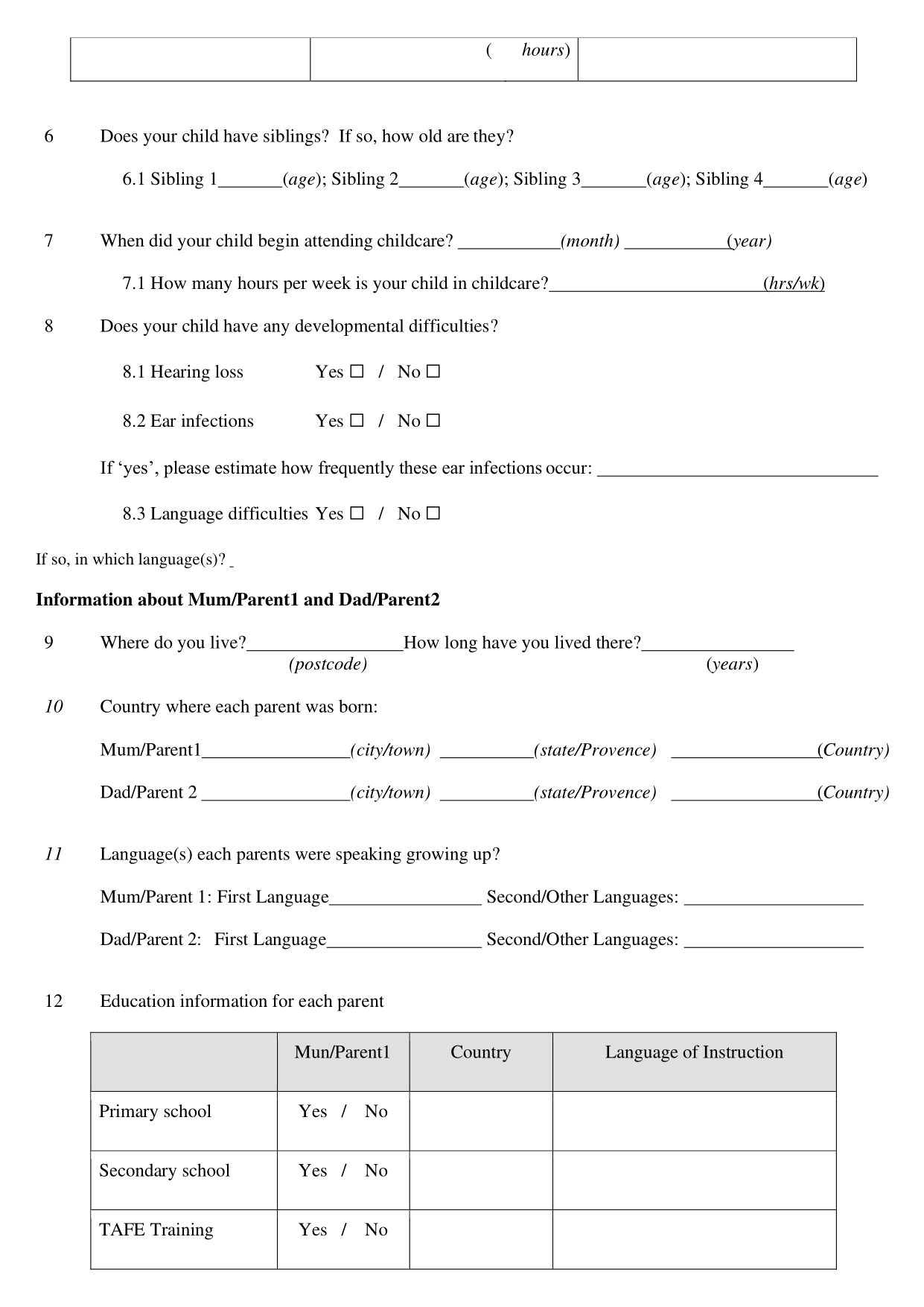

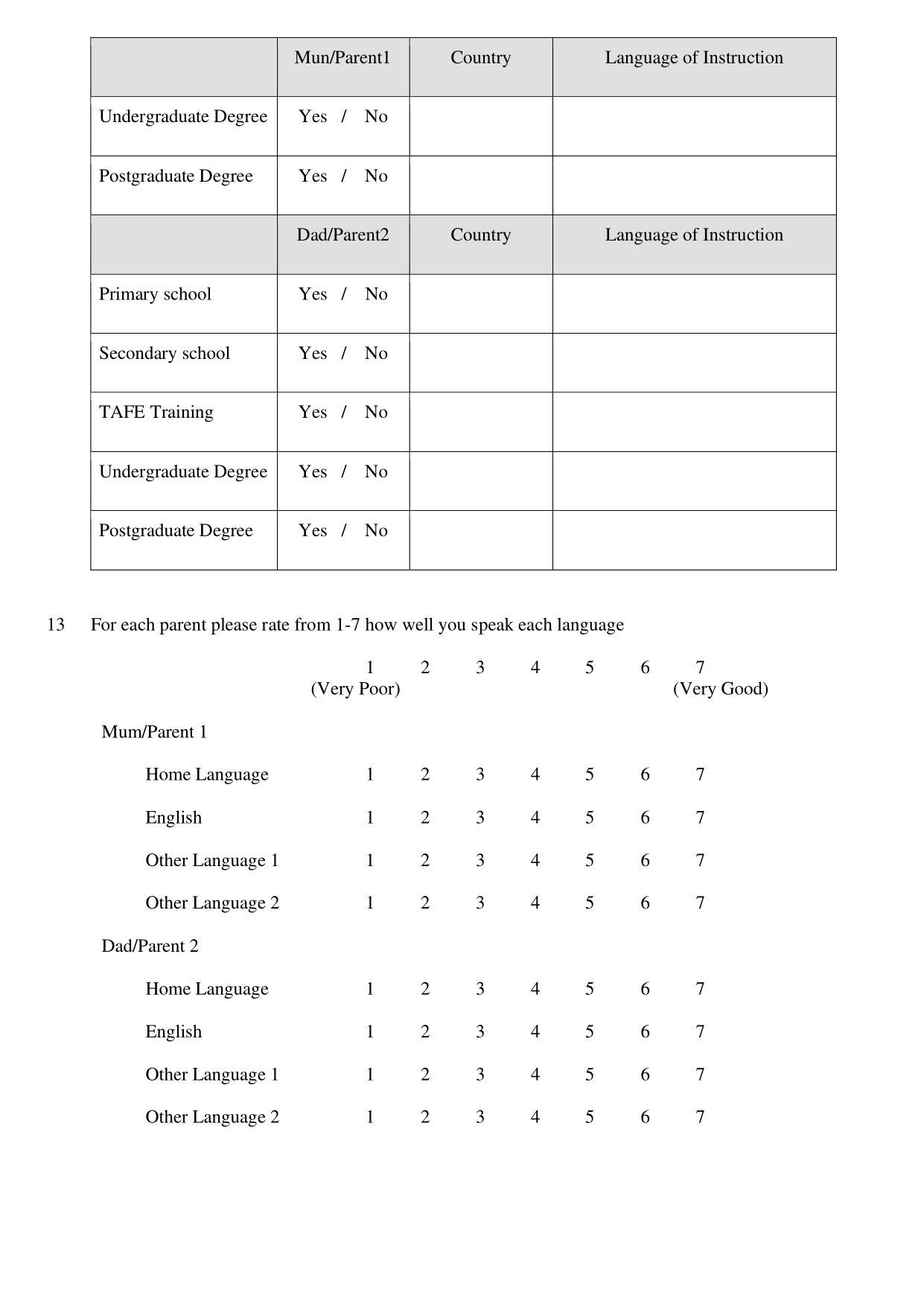

Supplement: Supplementary file 1 [file Table_1.docx]
