# Supplementary material for: Bilingual Mandarin-English preschoolers’ spoken narrative skills and contributing factors: A remote online story-retell study
Source: Front Psychol. 2022 Oct 14;13:797602. doi: 10.3389/fpsyg.2022.797602 (PMC9615547; doi:10.3389/fpsyg.2022.797602)
Supplement: Supplementary file 2 [file Table_2.docx]

# Appendix B. Macrostructure rubric for English

Based on Westerveld and Gillon (2008)


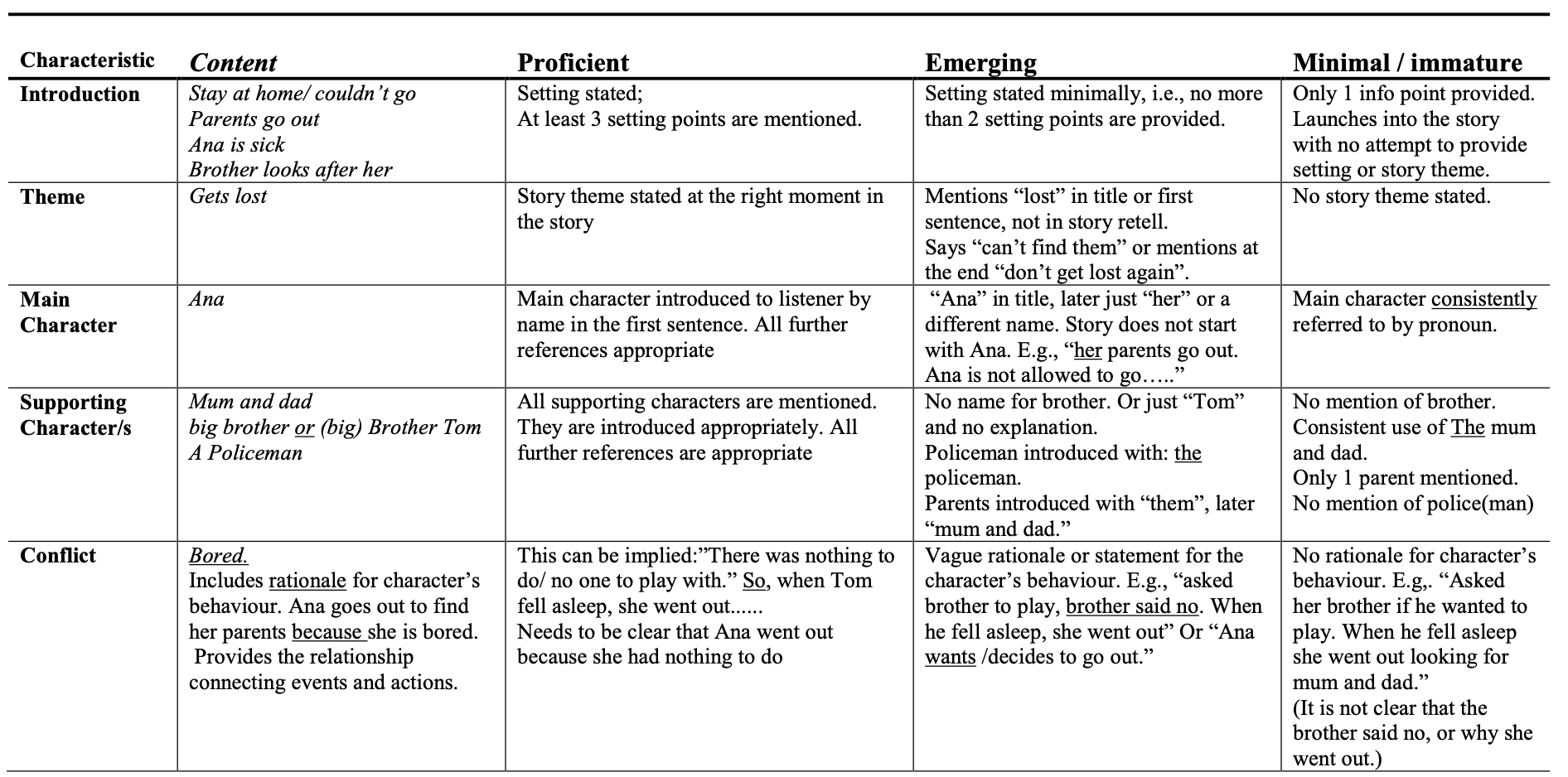


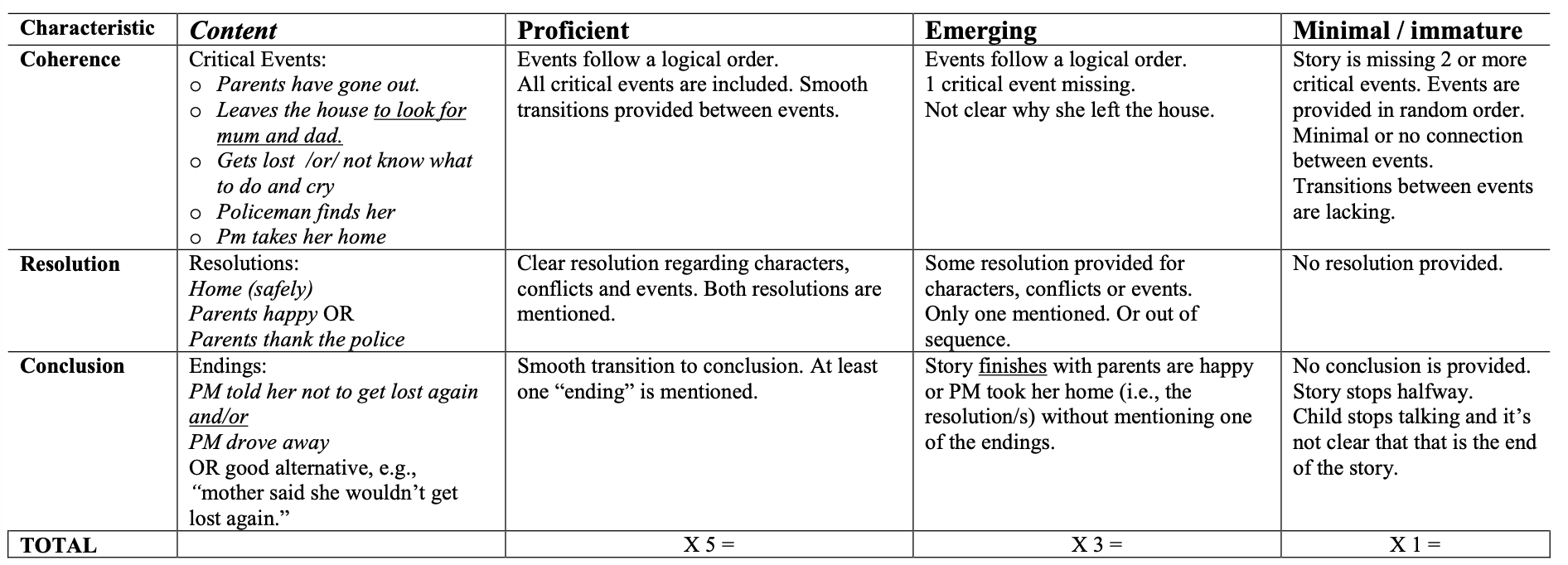


*Note:* The child was awarded points for each characteristic: 5 points if the child proficiently included the characteristic, 3 points if the skill was emerging, and 1 point if the child provided minimal or no information. The rubric includes specific scoring examples to promote easy and reliable scoring by other examiners. The scores were totalled to yield a total macrostructure score of English. As a result, the minimum score was 8 and the maximum score was 40.
