# Supplementary material for: Bilingual Mandarin-English preschoolers’ spoken narrative skills and contributing factors: A remote online story-retell study
Source: Front Psychol. 2022 Oct 14;13:797602. doi: 10.3389/fpsyg.2022.797602 (PMC9615547; doi:10.3389/fpsyg.2022.797602)
Supplement: Supplementary file 3 [file Table_3.docx]

**Appendix C.** **Macrostructure rubric for Mandarin**

| Characteristics | *Content* | Proficient | Emerging | Minimal/immature |
| --- | --- | --- | --- | --- |
| Introduction | *有一只小兔子*  */ 小兔子在路上走着*  *There was a bunny or*  *A bunny was walking on the road*  *小兔子拿着篮子*  *Holding a basket*  *有一颗苹果树*  There was an apple tree  小兔子想吃苹果  *The bunny wanted to eat apples* | Setting stated. At least 3 setting points are mentioned. | Setting stated minimally, i.e., no more than 2 setting points are provided. | Only 1 info point provided.  Launches into the story with no attempt to provide setting or story theme. |
| Theme | *小兔子摘苹果*  *The bunny picked apples* | Story theme stated at the right moment in the story. | Mentions “摘苹果” (pick apples) in title or first sentence, not in story retell. Says “想吃苹果/拿苹果/捡苹果” (wants to eat apples) or using other verbs “get/take” instead of “pick”. | No story theme stated. |
| Main character | *小兔子*  *Bunny* | Main character introduced to listener by name in the first sentence. All further references appropriate | “小兔子” (bunny) in title, later just “it” or a different name.  Story does not start with “小兔子(bunny)”. E.g., “路上有一棵苹果树” (There’s an apple tree on the road). | Main character consistently referred to by pronoun “它” (it). |
| Supporting character | *小刺猬*  Little hedgehog | The supporting character is mentioned by name “小刺猬” (Little hedgehog) and introduced appropriately. All further references are appropriate. | No name for “小刺猬” (Little hedgehog). Or using “小动物” (little animal) or “it” and no explanation. | No mention of the supporting character. |
| Conflict | *篮子里苹果太多了*  *There were too many apples in the basket.*  *Includes rationale for character’s behavior. The tree branch was broken, and apples and bunny fell down because there were too many apples in the basket. Provides the relationship connecting events and actions.* | This can be implied: “The basket was too heavy.” So, the tree branch was broken, and the bunny fell down.  Needs to be clear that the bunny fell down because there were too many in the basket. | Vague rationale or statement for the character’s behavior. E.g., “小兔子摘了好多苹果，然后它就掉下来了” (The bunny picked many apples and it fell down) 。 | No rationale for character’s behavior. E.g., ““小兔子摘了苹果放到篮子里，它掉下来了” (The bunny picked apples and put them in the basket. It fell down to the ground). |
| Coherence | Critical Events:   1. *小兔子看到一颗苹果树 / 小兔子想吃苹果*   *The bunny saw an apple tree*   1. *小兔子摘苹果*   *The bunny picked apples*   1. *小兔子摔下来了*   *The bunny fell down*   1. *苹果掉下来了/满地都是*   *Apples fell down*   1. *小刺猬把苹果捡起来/放进篮子里*   *The little hedgehog picked up apples / put apples back into basket* | Events follow a logical order. All critical events are included. Smooth transitions provided between events. | Events follow a logical order. 1 critical event missing. Not clear why the bunny picked apples. | Story is missing 2 or more critical events.  Events are provided in random order. Minimal or no connection between events. Transitions between events are lacking. |
| Resolution | *小刺猬帮小兔子把苹果捡起来，放进篮子里*  *The little hedgehog helped the bunny to pick up apples and put them back into the basket.* | Clear resolution regarding characters (*bunny and hedgehog*), conflicts (*apples on the ground*) and events (*Little hedgehog put them back to baske*t). | Some resolution provided for characters, conflicts, or events. | No resolution provided. |
| Conclusion | *小兔子很开心 / 小兔子笑了*  *The bunny was very happily*  *Or*  *The bunny smiled* | Smooth transition to conclusion.  At least one “ending” is mentioned. | Story finishes with little hedgehog put apples back (i.e., the resolution) without mentioning the ending. | No conclusion is provided. Story stops halfway. Child stops talking and it’s not clear that it’s the end of the story. |
| Total |  | X5= | X3= | X1= |
