# Supplementary material for: Bilingual Mandarin-English preschoolers’ spoken narrative skills and contributing factors: A remote online story-retell study
Source: Front Psychol. 2022 Oct 14;13:797602. doi: 10.3389/fpsyg.2022.797602 (PMC9615547; doi:10.3389/fpsyg.2022.797602)
Supplement: Supplementary file 4 [file Table_4.docx]

# Appendix D. Microstructure rubric for English

| Domain | English elements | Examples | 0 | 1 | 2 | 3(+) |
| --- | --- | --- | --- | --- | --- | --- |
| Phrase | Passive structure | She **got lost**.  The policeman told Ana not to **get lost** again.  Are you **called** Ana? |  |  |  |  |
|  | Locative phrase | Ana’s mon and dad went fishing **on the beach**.  She had to stay **at home**.  Ana walked **towards the beach**.  She stopped **outside at dairy.**  Then Ana felt a little tap **on her shoulder**.  The policeman took Ana home **in the police car**. |  |  |  |  |
|  | Temporal phrase | **One Saturday morning**  Ana had been sick **all week**  **When** **Tom fell asleep**, she decided to go looking for her mom...  She kept walking **until it got dark**. |  |  |  |  |
| Modifier  (Type) | Adjective | Ana had been **sick** **all** week.  **big** brother Tom.  She quietly opened the **front** door.  She kept walking until it got **dark**.  Ana have been **lost**  **Never go lost again**  Ana felt a **little** tap on her shoulder.  giving him a **big** smile  Mom and dad were very **happy** to see Ana |  |  |  |  |
|  | Adverb | Tom fell **asleep**  She **quietly** opened the front door and went **outside.**  Ana walked **towards** the beach.  She stopped **outside** at dairy  She looked **around**.  bringing her **home** **safely**  Then he smiled and drove **away**.  **There**  **Back home** |  |  |  |  |
|  | Negation | He said **no**  Ana **didn’t** know what to do.  The policeman told Ana **not to** get lost again |  |  |  |  |
| Nominal  (Type) | Personal pronoun | **She** looked around and saw a policeman  **She** had to stay at home with her big brother Tom.  **She** asked Tom if **he** wanted to play with **her**.  **He** said, “**I** want to read a Sports magazine”.  **She** decided to go looking for her mom and dad.  **She** quietly opened the front door.  **She** got lost.  **She** started to cry.  **She** looked around and saw a policeman.  Are **you** Ana?  Took **her** home  Then **he** smiled and drove away.  Thank **you**! |  |  |  |  |
| Verb  (Type) | Copula / Auxiliary / Model verb have to | It **was getting** dark.  She **was** still cry**ing.**  **Are** you Ana?  Mom and dad **were** very happy to see Ana.  **Have** **been** lost  Ana **had** **been** sick all week  Did not? |  |  |  |  |
|  | Irregular past tense verb | Ana’s mon and dad **went** fishing on the beach.  She quietly opened the front door and **went** outside.  When Tom **fell** asleep  She **kept** walking until it got dark.  Then Ana **felt** a little tap on her shoulder.  She looked around and **saw** a policeman.  he **said**  The policeman **took** Ana home.  The policeman **told** Ana not to get lost again  Then he smiled and **drove** away. |  |  |  |  |
|  | Regular past tense verb | She **started** to cry.  She **asked** Tom if he **wanted** to play with her.  She **decided** to go looking for her mom and dad.  She quietly **opened** the front door.  Ana **walked** towards the beach.  She **started** to cry.  She **stopped** outside at dairy.  She **looked** around.  They **thanked** the policeman.  Then he s**miled.** |  |  |  |  |
