# Supplementary material for: Bilingual Mandarin-English preschoolers’ spoken narrative skills and contributing factors: A remote online story-retell study
Source: Front Psychol. 2022 Oct 14;13:797602. doi: 10.3389/fpsyg.2022.797602 (PMC9615547; doi:10.3389/fpsyg.2022.797602)
Supplement: Supplementary file 5 [file Table_5.docx]

# Appendix E. Microstructure rubric for Mandarin

| Domain | Mandarin elements | Examples | 0 | 1 | 2 | 3(+) |
| --- | --- | --- | --- | --- | --- | --- |
| Phrase | “ba” structure  (Mandarin active structure with a special word order of “subject + ba + object + verb”) | **把** 摘 下来 的 苹果 都 放进 了 篮子 里面。  (ba) put all the picked apples into the basket.  **把** 树枝 压断 了  (ba) branch crushed.  它 帮 小兔子 **把** 苹果 捡 了 起来。  It helped (ba) the bunny to pick up apples. |  |  |  |  |
|  | Locative phrase | **在 路上**on the road  它 **手 里**In its hand  **远方** 有 一 颗 苹果 树  There was an apple tree in the distance  篮子 **里面** Inside the basket  **篮子 里 的** 苹果 放 得 太 多 了  There were too many apples in the basket.  小兔子 就 **从 树上** 摔 了 下来  The bunny fell off the tree  苹果 也 滚 得 **满 地 都是**  Apples rolled all over the ground  它 帮 小兔子 把 **地上 的** 苹果 都 捡 了 起来，放 到了 **篮子 里面**。  It helped the bunny pick up all the apples on the ground and put them into the basket. |  |  |  |  |
|  | Temporal phrase | **有 一 天** One day  **这时**，一 只 小 刺猬 刚好 经过。  At this time, a little hedgehog was just passing by. |  |  |  |  |
| Modifier | Adjective | **多**many  很 不 **高兴** very **unhappy**  **满** 地 都是 **all** over the ground  **开心**Happy |  |  |  |  |
|  | **Adverb** | **开心地** happily  **很** 想 / **好**想 want…very much  **很 不** 高兴 very not happy  **更 不** 开心more unhappy  **特别** 开心especially, very  **不** 开心 not happy  **不** 小心 not carefully  **太** 多 too much  **本来** originally  **都** all  **最** most  **再** again  **就** then; right after  **刚** just now  **还** still |  |  |  |  |
| Nominal | Classifier (number + classifier + noun)  (score for tokens) | 一 **只** 小兔子  one (classifier) bunny  一 **只** 篮子  one (classifier) basket  一 **颗** 苹果树  one (classifier) apple tree  一 **只** 小刺猬  one (classifier) hedgehog |  |  |  |  |
|  | Personal pronoun  (score for tokens) | **它** 手里 拿 着 一 只 篮子  It was holding a basket in its hand.  **它** 看 到 远方 有 一 颗 苹果树  It saw an apple tree in the distance  **它** 爬 上 了 苹果树  It climbed up the apple tree  **它** 帮 小兔子 It helped the bunny |  |  |  |  |
| Verb +  Aspect markers | Progressive aspect /imperfective  在/着  (zai/zhe) | 一 只 小兔子 在 路上 走 **着**  A bunny was walking on the road  它 手里 拿 **着** 一 只 篮子  It was holding a basket in its hand |  |  |  |  |
|  | Perfective aspect  了/过(le/guo) | 它 爬上 **了** 苹果树  It climbed up the apple tree.  放 进 **了** 篮子 里面  Put into the basket  小兔子 就 从 树 上 摔 **了** 下来  The bunny fell off from the tree  小兔子 摔 到 **了** 地上  The bunny fell down to the ground  把 摘 下来 的 苹果 都 放进 **了** 篮子 里面  Put all the picked apples into the basket.  放 到 **了** 篮子 里面  (apples have been) put into the basket. |  |  |  |  |
|  | Resultative (a modifier following a verb showing the result of the action) | 它 看 **到/见** 远方 有 一 颗 苹果树  It saw an apple tree in the distance  它 爬上 **了** 苹果树  It climbed up the apple tree.  篮子 里 的 苹果 放 **得 太多 了**  Too many apples were put inside the basket.  把 树枝 压 **断**了  The tree branch was broken **apart**  摔 **倒** 了Fall **upside down**  摔 **到** 了 地上 fell down to the ground  摔 **下来**Fall down  苹果 也 滚 **得 满 地 都是**  Apples also rolled all over the ground.  它 帮 小兔子 把 苹果 捡 了 **起来**  It helped (ba) the bunny to pick up apples  放 **进** / **到** 了 篮子 里面  Put all the picked apples into the basket. |  |  |  |  |
