# Supplementary material for: Bilingual Mandarin-English preschoolers’ spoken narrative skills and contributing factors: A remote online story-retell study
Source: Front Psychol. 2022 Oct 14;13:797602. doi: 10.3389/fpsyg.2022.797602 (PMC9615547; doi:10.3389/fpsyg.2022.797602)
Supplement: Supplementary file 6 [file Table_6.docx]

# Appendix F. Pairwise comparisons between macrostructure elements

English:

| Element 1 | Element 2 | *t* | Adjusted  *p*-value | Significance |
| --- | --- | --- | --- | --- |
| Coherence | Conclusion | -2.24 | 0.079 | Not significant |
| Coherence | Conflict | 0.52 | 0.661 | Not significant |
| Coherence | Introduction | -0.27 | 0.835 | Not significant |
| Coherence | Main character | -1.63 | 0.195 | Not significant |
| Coherence | Resolution | -3.27 | 0.013 | * |
| Coherence | Supporting character(s) | -1.83 | 0.142 | Not significant |
| Coherence | Theme | -3.33 | 0.013 | * |
| Conclusion | Conflict | 3.04 | 0.020 | * |
| Conclusion | Introduction | 2.13 | 0.093 | Not significant |
| Conclusion | Main character | 0.57 | 0.649 | Not significant |
| Conclusion | Resolution | -0.70 | 0.586 | Not significant |
| Conclusion | Supporting character(s) | 1.16 | 0.372 | Not significant |
| Conclusion | Theme | 0.21 | 0.841 | Not significant |
| Conflict | Introduction | -0.68 | 0.586 | Not significant |
| Conflict | Main character | -2.94 | 0.020 | * |
| Conflict | Resolution | -3.62 | 0.007 | ** |
| Conflict | Supporting character(s) | -2.99 | 0.020 | * |
| Conflict | Theme | -2.24 | 0.079 | Not significant |
| Introduction | Main character | -1.44 | 0.250 | Not significant |
| Introduction | Resolution | -2.98 | 0.020 | * |
| Introduction | Supporting character(s) | -1.56 | 0.211 | Not significant |
| Introduction | Theme | -1.90 | 0.137 | Not significant |
| Main character | Resolution | -0.89 | 0.533 | Not significant |
| Main character | Supporting character(s) | 0.70 | 0.586 | Not significant |
| Main character | Theme | -0.20 | 0.841 | Not significant |
| Resolution | Supporting character(s) | 1.83 | 0.142 | Not significant |
| Resolution | Theme | 0.72 | 0.586 | Not significant |
| Supporting character(s) | Theme | -0.77 | 0.586 | Not significant |

**p* < 0.05, ** *p* < 0.01, ****p* < 0.001

Mandarin:

| Element 1 | Element 2 | *t* | Adjusted  *p*-value | Significance |
| --- | --- | --- | --- | --- |
| Coherence | Conclusion | -3.04 | 0.012 | * |
| Coherence | Conflict | -1.00 | 0.410 | Not significant |
| Coherence | Introduction | 0.00 | 1.000 | Not significant |
| Coherence | Main character | -5.21 | <0.001 | *** |
| Coherence | Resolution | -5.10 | <0.001 | *** |
| Coherence | Supporting character(s) | -3.94 | 0.002 | ** |
| Coherence | Theme | -5.60 | <0.001 | *** |
| Conclusion | Conflict | 1.71 | 0.150 | Not significant |
| Conclusion | Introduction | 3.04 | 0.012 | * |
| Conclusion | Main character | -2.37 | 0.044 | * |
| Conclusion | Resolution | -1.42 | 0.228 | Not significant |
| Conclusion | Supporting character(s) | -1.67 | 0.152 | Not significant |
| Conclusion | Theme | -1.83 | 0.124 | Not significant |
| Conflict | Introduction | 1.00 | 0.410 | Not significant |
| Conflict | Main character | -4.00 | 0.002 | ** |
| Conflict | Resolution | -2.94 | 0.014 | * |
| Conflict | Supporting character(s) | -2.67 | 0.025 | * |
| Conflict | Theme | -3.04 | 0.012 | * |
| Introduction | Main character | -4.36 | 0.001 | *** |
| Introduction | Resolution | -4.34 | 0.001 | *** |
| Introduction | Supporting character(s) | -3.45 | 0.006 | ** |
| Introduction | Theme | -5.60 | <0.001 | *** |
| Main character | Resolution | 0.72 | 0.577 | Not significant |
| Main character | Supporting character(s) | 0.62 | 0.628 | Not significant |
| Main character | Theme | 0.49 | 0.708 | Not significant |
| Resolution | Supporting character(s) | -0.27 | 0.835 | Not significant |
| Resolution | Theme | -0.33 | 0.816 | Not significant |
| Supporting character(s) | Theme | 0.00 | 1.000 | Not significant |

**p* < 0.05, ** *p* < 0.01, ****p* < 0.001
