# Supplementary material for: Bilingual Mandarin-English preschoolers’ spoken narrative skills and contributing factors: A remote online story-retell study
Source: Front Psychol. 2022 Oct 14;13:797602. doi: 10.3389/fpsyg.2022.797602 (PMC9615547; doi:10.3389/fpsyg.2022.797602)
Supplement: Supplementary file 7 [file Table_7.docx]

# Appendix G. Proportion of Model-Like Fine-grained Microstructure Produced

| English | | | | | | | |
| --- | --- | --- | --- | --- | --- | --- | --- |
| Domain | **Item** | **0-3 Frequency**  **Mean (SD)** | **Model Story** | **Percentage** | **Raw Frequency**  **Mean (SD)** | **Model Story** | **Percentage** |
| Modifier | Adjective | 2.05 (1.23) | 3 | 68.33% | 2.45 (1.82) | 10 | 24.50% |
|  | Adverb | 1.85 (0.99) | 3 | 61.67% | 2.25 (1.62) | 8 | 28.13% |
|  | Negation | 0.55 (0.60) | 3 | 18.33% | 0.55 (0.60) | 3 | 18.33% |
|  | **Average** | **1.48 (0.74)** | **3** | 49.33% | **1.75 (1.12)** | **7** | 25.00% |
| Nominal | Pronoun | 2.15 (1.04) | 3 | 71.67% | 2.80 (1.82) | 6 | 46.67% |
|  | **Average** | **2.15 (1.04)** | **3** | 71.67% | **2.80 (1.82)** | **6** | 46.67% |
| Phrase | Locative phrase | 1.10 (1.21) | 3 | 36.67% | 1.40 (1.82) | 6 | 23.33% |
|  | Passive phrase | 0.35 (0.49) | 1 | 35.00% | 0.35 (0.49) | 3 | 11.67% |
|  | Temporal phrase | 1.00 (1.08) | 3 | 33.33% | 1.10 (1.29) | 4 | 27.50% |
|  | **Average** | **0.82 (0.69)** | **2.33** | 35.19% | **0.95 (0.97)** | **4.33** | 21.94% |
| Verb | Copula & Auxiliary | 1.60 (1.19) | 3 | 53.33% | 1.7 (1.38) | 4 | 42.50% |
|  | Irregular past tense | 2.15 (1.14) | 3 | 71.67% | 2.80 (1.96) | 10 | 28.00% |
|  | Regular past tense | 1.15 (1.27) | 3 | 38.33% | 1.60 (2.16) | 10 | 16.00% |
|  | **Average** | **1.63 (1.05)** | **3** | 54.33% | **2.03 (1.68)** | **8** | 25.38% |
| Total |  |  |  |  | **17.00(0.87)** | **64** | **26.56%** |

| Mandarin | | | | | | | |
| --- | --- | --- | --- | --- | --- | --- | --- |
| Domain | **Item** | **0-3 Frequency** | **Model Story** | **Proportion** | **Raw Frequency** | **Model Story** | **Proportion** |
| Modifier | Adjective | 1.95 (1.00) | 3 | 65.00% | 2.05 (1.19) | 3 | 68.33% |
|  | Adverb | 2.10 (1.12) | 3 | 70.00% | 2.45 (1.54) | 7 | 35.00% |
|  | Classifier | 1.90 (0.97) | 3 | 63.33% | 1.90 (0.97) | 4 | 47.50% |
|  | **Average** | **1.98 (0.69)** | **3** | 66.00% | **2.13 (0.85)** | **4.67** | 45.61% |
| Nominal | Pronoun | 2.30 (1.03) | 3 | 76.67% | 4.00 (2.96) | 4 | 100.00% |
|  | **Average** | **2.30 (1.03)** | **3** | 76.67% | **4.00 (2.96)** | **4** | 100.00% |
| Phrase | Locative phrase | 1.45 (1.10) | 3 | 48.33% | 1.45 (1.10) | 8 | 18.13% |
|  | Ba structure | 0.60 (0.60) | 3 | 20.00% | 0.60 (0.60) | 3 | 20.00% |
|  | Temporal phrase | 0.35 (0.49) | 2 | 17.50% | 0.35 (0.49) | 2 | 17.50% |
|  | **Average** | **0.80 (0.45)** | **2.67** | 29.96% | **0.80 (0.45)** | **4.33** | 18.48% |
| Verb | Perfective aspect marker | 2.35 (0.81) | 3 | 78.33% | 3.55 (2.16) | 6 | 59.17% |
|  | Progressive aspect marker | 0.55 (0.60) | 2 | 27.50% | 0.55 (0.60) | 2 | 27.50% |
|  | Resultative aspect marker | 2.15 (0.99) | 3 | 71.67% | 2.55 (1.50) | 8 | 31.88% |
|  | **Average** | **1.68 (0.59)** | **2.67** | 62.92% | **2.22 (1.09)** | **5.33** | 41.65% |
| Total |  |  |  |  | **19.45(1.25)** | **47** | **41.38%** |
