# Supplementary material for: Bilingual Mandarin-English preschoolers’ spoken narrative skills and contributing factors: A remote online story-retell study
Source: Front Psychol. 2022 Oct 14;13:797602. doi: 10.3389/fpsyg.2022.797602 (PMC9615547; doi:10.3389/fpsyg.2022.797602)
Supplement: Supplementary file 8 [file Table_8.docx]

# Appendix H. Pairwise comparisons between microstructure domains

English:

| Domain 1 | Domain 2 | *t* | Adjusted  *p*-value | Significance |
| --- | --- | --- | --- | --- |
| Modifier | Nominal | -3.08 | 0.007 | ** |
| Modifier | Phrase | 4.66 | <0.001 | *** |
| Modifier | Verb | -0.87 | 0.394 | Not significant |
| Nominal | Phrase | 7.18 | <0.001 | *** |
| Nominal | Verb | 3.94 | 0.001 | *** |
| Phrase | Verb | -5.44 | <0.000 | *** |

**p* < 0.05, ** *p* < 0.01, ****p* < 0.001

Mandarin:

| Domain 1 | Domain 2 | *t* | Adjusted  *p*-value | | Significance |
| --- | --- | --- | --- | --- | --- |
| Modifier | Nominal | -1.33 | 0.200 | Not significant | |
| Modifier | Phrase | 9.17 | <0.001 | *** | |
| Modifier | Verb | 2.54 | 0.024 | * | |
| Nominal | Phrase | 5.63 | <0.001 | *** | |
| Nominal | Verb | 2.76 | 0.019 | * | |
| Phrase | Verb | -5.90 | <0.001 | *** | |

**p* < 0.05, ** *p* < 0.01, ****p* < 0.001
